# Evaluation of Pharmacist-Developed Educational Leaflets for Women’s Health: A Pre–Post Study of Knowledge and Perceived Usefulness

**DOI:** 10.3390/pharmacy14010029

**Published:** 2026-02-05

**Authors:** Weronika Guzenda, Zuzanna Berdzińska, Piotr Przymuszała, Olga Sierpniowska, Magdalena Jasińska-Stroschein, Magdalena Waszyk-Nowaczyk

**Affiliations:** 1 Pharmacy Practice and Pharmaceutical Care Division, Chair and Department of Pharmaceutical Technology, Poznan University of Medical Sciences, 3 Rokietnicka Street, 60-806 Poznan, Poland; olga.sierpniowska@gmail.com; 2 Students’ Pharmaceutical Care Group, Pharmacy Practice and Pharmaceutical Care Division, Chair and Department of Pharmaceutical Technology, Poznan University of Medical Sciences, 3 Rokietnicka Street, 60-806 Poznan, Poland; 85335@student.ump.edu.pl; 3 Department of Medical Education, Poznan University of Medical Sciences, 7 Rokietnicka Street, 60-806 Poznan, Poland; pprzymuszala@ump.edu.pl; 4 Department of Biopharmacy, Medical University of Lodz, 1 Muszynskiego Street, 90-151 Lodz, Poland; magdalena.jasinska-stroschein@umed.lodz.pl

**Keywords:** pharmaceutical care, written educational materials, pharmacist, medical education, women’s health

## Abstract

Background: Written educational materials are widely used in community pharmacies to support patient education, and available evidence suggests their effectiveness in improving short-term knowledge. However, there remains a need for well-documented, practice-oriented evaluations of pharmacist-developed materials in real-world community pharmacy settings. The aim of this study was to evaluate the immediate impact of a pharmacist-developed educational leaflet on women’s health knowledge and its perceived usefulness, clarity, and acceptability. Methods: This study evaluated pharmacist-developed educational leaflets addressing women’s health topics using a pre–post study design. The study was conducted in Poland and involved 266 adult women. All participants completed a five-question knowledge test before and immediately after reading the educational leaflet, followed by a self-assessment of perceived usefulness, clarity, and visual appeal. Descriptive statistics were performed to summarize the results. Results: A statistically significant increase in knowledge was observed after exposure to the educational material, with mean scores rising from 2.8 ± 1.2 to 4.6 ± 0.7 (out of 5, *p* < 0.001). The greatest improvements were noted in topics related to sexually transmitted infection self-testing and pregnancy testing. Most participants rated the leaflet as useful, comprehensible, attractive, and engaging, with higher ratings reported among younger and better-educated respondents. Conclusions: Pharmacist-developed educational leaflets can support short-term knowledge acquisition and are perceived positively by women across age groups. These findings highlight the potential role of community pharmacies in delivering accessible written health education, while underscoring the need for future studies to assess long-term knowledge retention, behavioral outcomes, and topic-specific, targeted materials.

## 1. Introduction

Pharmacies are highly accessible health facilities where not only medications but also professional counsel and health promotion are provided by pharmacists. Pharmacists are highly trusted by the public and possess extensive knowledge of pharmacotherapy, human physiology, and disease prevention [1,2]. In Poland and other countries, where primary care physicians may be less available, pharmacists now undertake responsibilities like patient education and preventive care [3,4].

This enlarging role of the pharmacist is in harmony with global health strategies to extend access to and quality of prevention services outside the normal clinical setting [5,6]. Pharmacists are ideally positioned to deliver such services due to their regular contact with the community, professional credibility, and proficiency in both the pharmacological and lifestyle sides of healthcare.

In Poland, public health outreach efforts to more actively incorporate pharmacists are being promoted by legislative reforms and the broadening mission of pharmaceutical care [7]. The changes hold out the promise of new roles for pharmacists as frontline health advisors and educators, particularly in underserved communities.

Women’s health remains a continuing public health issue. Preventive screening such as Pap smears, HPV testing, and mammograms is recommended but low in uptake, particularly among less educated women or women with lower socioeconomic status [8,9]. Young women, on the other hand, face mounting lifestyle-related risks of alcohol consumption, smoking, and physical inactivity [10]. Due to these problems, health education according to women’s needs should be implemented [11,12].

Written educational materials, such as leaflets and brochures, have been used in healthcare for decades to help patients learn and manage their health. Since the 1970s, many studies have looked at how these materials improve patients’ understanding of diseases, treatments, and preventive measures for various conditions, from acute infections to chronic and cardiovascular diseases [13,14,15].

Systematic reviews have consistently shown that educational leaflets can improve patient knowledge, understanding and satisfaction in the short term, particularly when the materials are clearly written and tailored to the target group [14,15,16,17,18]. However, evidence on long-term retention and sustained behavior change remains inconsistent and is highly dependent on content quality, readability, health literacy, and contextual factors [19,20,21,22,23]. Recent reviews indicate that poorly designed educational materials often have limited effectiveness, particularly among individuals with low health literacy [18,21]. Unlike many previous studies focusing solely on short-term knowledge outcomes of generic patient information materials, the present study evaluates a pharmacist-developed leaflet within the real-world context of community pharmacy practice, combining knowledge assessment with users’ perceived usefulness and clarity [15,16].

In recent years, there has been growing attention on using plain language, effective visual design, and a focus on users when creating written materials. This is particularly important for addressing misinformation and reducing health disparities. Still, there is limited evidence regarding educational leaflets developed by pharmacists and their evaluation in community pharmacy settings, especially in women’s health [17,18].

Educational products dispensed by pharmacists, in the form of leaflets, can be good tools for health awareness and encouraging healthy behaviors [20]. The efficacy of such materials and their impact on women’s knowledge were to be determined by the current study. The aim of this study was to evaluate the immediate impact of a pharmacist-developed educational leaflet on women’s health knowledge and its perceived usefulness, clarity, and acceptability.

## 2. Materials and Methods

Between September and December 2024, an internet-based survey was distributed to adult women aged 18 years and older residing in Poland. The survey was conducted in three parts: a pre-intervention knowledge test, reading the educational leaflet, and a post-intervention knowledge test. To assess the clarity and comprehensibility of the educational leaflet and survey questions, a pilot test was conducted with 10 women prior to the main study. Feedback from the pilot was used to refine the wording and layout of the materials. Data collected during the pilot phase were not included in the final analysis.

The patient education leaflet was developed in 2024 in the Department of Practical Pharmacy and Pharmaceutical Care at Poznan University of Medical Sciences within the framework of the academic project of patient education under pharmacists’ supervision. The educational leaflet was developed by a team of pharmacists, including community pharmacists with direct patient-care experience and academic pharmacists involved in pharmacy education and research. It was intended to provide understandable, evidence-based information for the benefit of women’s health literacy and preventive behavior.

The development followed a standard process: (1) topic choice from the most common questions pharmacists are asked in community practice (contraception, pregnancy testing, medication safety, and STI prevention); (2) preparation of draft text following WHO and CDC health literacy guidelines [24,25,26]; (3) review by experts for clinical accuracy and plain language use; and (4) professional graphic design in a tri-fold A4 format (two-sided, full-color) with icons, pictograms, and short sections emphasizing key messages.

The leaflet design included bold headers, bullet-pointed advice, and infographic summaries for each topic. Visual coherence (blue–pink color scheme, sans-serif typeface, high-contrast text) was maintained to optimize readability and beauty.

The educational leaflet consisted of four main sections:

(1) Hormonal contraception and its correct use,
(2) Safe medication use during pregnancy and breastfeeding,
(3) Basics of pregnancy testing and interpretation of results,
(4) Prevention and home-testing of sexually transmitted infections (STIs).

Each section included short text, infographics, and QR codes connecting to reliable sources of medical information.

The educational pamphlet utilized in this study is presented in the Supplementary Materials (File S1). It was the core intervention medium to improve women’s knowledge on health and was distributed digitally during the survey. The content and visual presentation were maximized for readability and interest according to plain-language and health communication principles.

To ensure the consistency and conciseness of the educational leaflet, its creation process involved consultation with formulators of health communication and pharmacists. The language was rendered simple to read, adhering to plain language guidelines, and presentation featured illustrations and bolded terms for ease of reading. Participants were recruited via online health discussion forums, women’s social media sites, and newsletters circulated within networks of pharmacies. An educational leaflet was digitally distributed as part of the online survey, and participants completed a pretest, read the leaflet, and completed a posttest during the same survey session. No in-person recruitment or direct pharmacist–patient interaction was conducted. Although the leaflet was designed for both digital and printed use in community pharmacy settings, in this study it was delivered exclusively in digital form as part of the online survey.

The post-intervention knowledge test was completed immediately after reading the educational leaflet during the same survey session. The knowledge test consisted of five questions covering four focus areas: hormonal contraception (one question), pregnancy testing (two questions), sexually transmitted infection (STI) self-testing (one question), and medication safety during pregnancy (one question). The same set of questions was used in both the pre- and post-intervention assessments. Objective knowledge was measured using a test, while perceived knowledge was assessed using self-assessment questions. Each knowledge question was scored dichotomously. A correct answer was awarded 1 point. Incorrect answers, unanswered questions, partially correct answers, and “I don’t know” responses were scored 0 points. Each participant’s overall knowledge score was calculated by summing the correct answers to all five questions, resulting in a total score ranging from 0 to 5. Knowledge scores were analyzed both at the individual question level and based on the total knowledge score. The original knowledge test is available in the Supplementary Materials (File S2).

The respondents also completed a satisfaction questionnaire assessing the visual appeal, clarity, usability, and interest of the leaflet. This was a 15-item Likert-type questionnaire with some open-ended questions where respondents could leave spontaneous remarks about the aesthetics, clarity, tone, and perceived usefulness of the leaflet. Demographic data including level of education, age, and residential area (urban or rural) were collected to enable comparison of subgroup findings. The questionnaire consisted primarily of closed-ended questions. Open-ended questions were limited to optional comments regarding proposed changes to the leaflet and additional comments. Responses to these open-ended questions were analyzed using a descriptive qualitative approach, where comments were manually reviewed and grouped into broad thematic categories reflecting recurring suggestions or observations. The qualitative component was exploratory in nature and intended solely to complement the quantitative results. The original satisfaction survey questionnaire is provided in the Supplementary Materials (File S3).

STATISTICA 12 and STATA 14 were utilized for data analysis. Statistical tests included chi-square, Fisher’s exact test, Mann–Whitney U, and Kruskal–Wallis tests, and the significance level of α = 0.05 was defined.

Each respondent voluntarily consented to participate before participating in the study. The work was approved by the bioethics committee no. 579/24 at the Poznan University of Medical Sciences.

## 3. Results

### 3.1. Participant Characteristics

In total, 266 women took part in the research. Their demographic profiles are presented in Table 1. The majority of them were aged 25–34 years (41.1%), lived in large cities (36.5%), and were better educated (47.4%).

### 3.2. Knowledge Improvement

The highest baseline knowledge levels were observed for questions about hormonal contraception and medication safety during pregnancy, while the lowest baseline knowledge levels were observed for self-testing for STIs and interpretation of pregnancy tests. Improvements were observed across all topics after reading the leaflet, with the smallest relative change observed for questions about contraception, suggesting that these topics were already relatively well-known before the intervention.

After reading the educational leaflet, a statistically significant increase in participants’ knowledge was observed. Overall knowledge improved across all assessed topics, with particularly marked increases in areas related to the correct use of self-tests for sexually transmitted infections (STIs), followed by improved interpretation of pregnancy test results (Table 2).

By age, the biggest gain was achieved by the youngest cohort (18–24 years), an average increase of 2.0 points, compared with 1.4 for women aged 25–34 years and 0.9 for women aged over 35 years (Kruskal–Wallis, *p* = 0.018). Education also yielded the same result. Women with high education possessed the largest initial knowledge, but women with secondary education acquired the largest relative improvement, from 2.3 to 4.5.

**Figure 1:**
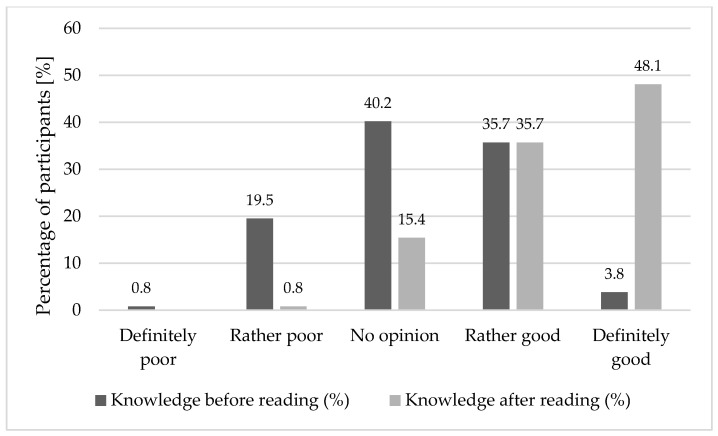
Self-assessment of respondents’ knowledge before and after reading educational materials on women’s health (n = 266).

Participants’ self-assessed knowledge increased following exposure to the leaflet, with a clear shift toward higher self-ratings and a reduction in uncertain responses. Figure 1 illustrates the improvement in the overall (composite) self-assessed knowledge score before and after reading the leaflet. Values represent the percentage of participants selecting each response category.

The pre- and post-test distribution of correct answers per question is presented in Supplementary Figure S1, serving as proof for statistically significant improvement in all areas of knowledge (*p* = 0.0001).

### 3.3. Satisfaction with the Leaflet

In total, 90.2% of respondents found the leaflet interesting, and 94.3% of them thought the leaflet was beautiful. About half of the interviewees (48.5%) stated that the leaflet “definitely interested them,” and another 41.7% were “rather interested,” bearing witness to its extensive popularity. Higher satisfaction scores were observed among younger participants (18–34 years) and those with higher education. A breakdown of the satisfaction ratings on all the evaluated dimensions (interest, aesthetics, transparency, understandability, and usefulness) is shown in Table 3. Table 3 presents the participants’ self-assessment of the perceived properties of the leaflet.

As Supplementary Figure S2 indicates, the level of interest declined slightly with age, from 4.6 for women aged 25–34 to 3.8 for those in the 55–64 age group. Level of education was also positively associated with the level of interest—the more highly educated averaged 4.5, compared with 3.7 among those with vocational education (Supplementary Figure S3).

The mean transparency ratings were lower with higher age, decreasing from 4.7 in women aged 25–34 years to 3.6 in those aged 55–64 years. Understanding also followed the same trend. As indicated in Table 4, differences in perceived understandability were observed between age groups; however, the pattern was not linear, with the highest ratings reported by participants aged 25–34 years, while both younger and older groups reported slightly lower scores. Values are presented as mean and median based on a five-point Likert scale. Understanding was most pronounced in those with higher education (mean = 4.7) compared to 4.1 among those who had vocational education (Supplementary Figure S4). The results for usability are similar—Supplementary Figure S5. Detailed distributions of scores across subgroups are provided in the Supplementary Materials (Supplementary Figures S1–S5).

### 3.4. Qualitative Feedback

Respondents valued plain language, sound advice, and visual aids (e.g., diagrams of contraception or pregnancy tests). The majority recommended dividing content into shorter topic-based leaflets or making them available digitally, for instance, through QR codes linking to brief videos. Some emphasized that pharmacies were seen as “more accessible” and “less intimidating” compared to clinics.

## 4. Discussion

This study supports the impact that educational materials developed by pharmacists can have on increasing women’s knowledge about reproductive health. The most significant impact was seen in those who had the lowest pre-interventional knowledge, as seen in other research on the effectiveness of plain-language materials [1,12]. Although the study assesses immediate post-intervention outcomes, its contribution lies in demonstrating how pharmacist-designed written materials can function as a practical, acceptable, and scalable educational tool in community pharmacies.

The visual display of the leaflet, structure, and accessibility made comprehension easier, as suggested by WHO and CDC [24] health communication principles. More importantly, participants who perceived the content as “clear” tended to achieve higher post-test scores, supporting research indicating that perceived clarity is a strong predictor of learning [6].

Younger participants demonstrated the greatest knowledge improvement and reported higher satisfaction with the leaflet. Results are in accord with overall literature on the value of relevant health information [11].

Open-ended answers corroborated this. Participants liked or preferred the tone, style, and convenience of accessing materials from pharmacies. These results concur with Kosycarz & Walendowicz’s research proving that personalization and availability increase engagement [12]. Participants also liked pharmacists as expert, accessible teachers [2,4,5].

Still, inequalities remain. Older women and women with lower educational attainment were less likely to rate the leaflet as easy to understand and tended to assign lower understandability scores. To address these differences, future interventions may benefit from combining written materials with oral counseling and multimedia content to enhance inclusivity and accessibility across diverse populations.

These findings also highlight the importance of transparency and reproducibility in pharmacist-led educational interventions. Transparency regarding the leaflet’s authorship, purpose, and design process is essential for replication. Future studies should publish visual examples or standardized templates to facilitate methodological reproducibility and cross-country comparison of pharmacist-led health education materials.

One notable finding was the impact of a single leaflet. Over 60% of women rated it as “definitely useful,” suggesting that even brief targeted education campaigns in pharmacies can contribute to public health. Earlier national policies suggest extending the role of pharmacists in prevention, especially among disadvantaged groups [3,7]. In the context of misinformation and declining screening rates [8,9], materials designed for use in community pharmacy settings may offer a less expensive, scalable, and consistent way.

In the broader public health context, the effectiveness of a single, well-designed educational leaflet is particularly noteworthy given the current challenges of misinformation, time constraints in healthcare, and declining participation in preventive programs. Women increasingly rely on informal or online sources of health information, which can be incomplete, inaccurate, or contradictory [27]. Community pharmacies, as trusted and easily accessible healthcare providers, can therefore play a key role in countering misinformation by offering concise, evidence-based educational materials that align with national and international recommendations [28].

Importantly, printed materials distributed through pharmacies can reach people who do not regularly utilize other parts of the healthcare system. This includes women who delay preventive screenings, avoid healthcare facilities due to anxiety or logistical barriers, or perceive their health problems as insufficiently serious to warrant a doctor’s visit [29]. In such contexts, educational leaflets can serve as a low-threshold intervention, raising awareness and prompting reflection on health behaviors without the need for immediate consultation with a physician [30].

The positive reception of the leaflet observed in this study supports the feasibility of incorporating similar materials into daily pharmacy practice. Even brief exposure to targeted educational content can contribute to improved awareness and preparedness for future health decision-making, especially when reinforced through repeated meetings or follow-up counseling.

In addition to the increased knowledge observed in this study, the results have a number of practical implications for community pharmacy practice and the broader organization of women’s health education. Community pharmacies are one of the most frequently visited points of contact with the healthcare system, often serving individuals who do not regularly use primary care or specialist consultations. Consequently, pharmacies provide low-threshold and easily accessible venues for health promotion, education, and preventive interventions, as demonstrated by recent analyses of the expanded role of community pharmacies in primary care systems [31].

Educational leaflets developed by pharmacists can complement existing healthcare services by answering common patient questions in a structured and evidence-based manner. Previous studies have shown that educational interventions delivered by pharmacists in community pharmacies can improve patient knowledge, medication safety, and health decision-making [32,33]. Written materials can further standardize the quality of information provided, ensuring adherence to clinical recommendations and reducing the variability associated with informal or ad hoc counseling, particularly in areas such as reproductive health, self-diagnosis, and medication use [32].

Integrating pharmacist-created educational materials into routine pharmacy workflows can also promote time-efficient interactions with patients. Given the increasing workload and time pressures in community pharmacies, written materials can serve as an extension of oral counseling, allowing patients to review information at their own pace and revisit key messages after the encounter [33]. This approach can be particularly valuable for sensitive or stigmatized topics, where patients may be reluctant to initiate discussion or may benefit from private reflection. From a healthcare systems perspective, the use of standardized educational materials in community pharmacies can contribute to improving health literacy at the population level. Systematic review evidence suggests that community pharmacists play a vital role in preventive care and chronic disease management through patient education and counseling, supporting broader public health goals. Although this study did not assess behavioral outcomes, a better understanding of health-related issues is widely recognized as essential for making informed health decisions and engaging in preventative care [34].

Finally, the development and evaluation of pharmacist-led educational tools aligns with ongoing efforts to strengthen the professional role of pharmacists beyond medication dispensing toward more patient-centered and preventative services. Actively engaging pharmacists in the creation and dissemination of educational materials can increase their visibility as credible sources of health information and facilitate closer integration of community pharmacies into multidisciplinary public health strategies [34,35].

### 4.1. Limitations

Several limitations of this study should be noted. First, the educational leaflet covered a wide range of women’s health topics relevant to different stages of life; therefore, not all sections were equally relevant to all age groups. In particular, content regarding contraception, pregnancy, or breastfeeding may have been less relevant to older participants. However, the primary goal of the study was to assess the clarity, comprehensibility, and perceived usefulness of the educational materials developed by pharmacists, not their individual clinical usefulness.

Second, the study employed a pre–post design with assessments conducted immediately after the intervention, which primarily addresses short-term knowledge acquisition. Although this approach is commonly used in educational research, the improvement in posttest scores may partially reflect exposure to the information rather than sustained understanding. Because the posttest was conducted immediately after reading the leaflet, conclusions regarding long-term knowledge retention or behavior change cannot be drawn.

Third, the study relied on a sample of motivated volunteers recruited online, which may have increased participants’ willingness to engage with the educational content and may limit the generalizability of the results. Furthermore, although pharmacists developed the leaflet, the research team did not include a dedicated clinical expert in women’s health, which may have influenced content prioritization.

Finally, some age subgroups were relatively small, reducing the reliability of subgroup analyses and suggesting that future studies might consider combining smaller categories to increase statistical power.

### 4.2. Future Research

Overall, the results support the value of pharmacist-created educational leaflets as effective tools for improving short-term knowledge and awareness of women’s health. However, because this study assessed immediate knowledge acquisition, future research should adopt a longitudinal design to assess long-term retention and behavioral outcomes. In particular, studies examining educational materials developed and actively delivered by pharmacists during face-to-face consultations in community pharmacies could provide deeper insights into their actual effectiveness. Furthermore, including surveys assessing participants’ intended actions after viewing the educational materials could help us better understand the mechanisms by which pharmacist-provided education influences preventive behaviors and health decision-making.

## 5. Conclusions

Educational materials developed by pharmacists for community pharmacy practice may serve as a useful tool for improving women’s health knowledge and may support preventive care through short-term knowledge enhancement. Clear, interesting, and scientifically accurate leaflets work well with patients and promote awareness. Pharmacists need to be reminded to incorporate such material into day-to-day counseling, particularly in community pharmacies where access is common.

As misinformation and health disparities, particularly among women of lower socioeconomic status, rise, precision-crafted interventions based on evidence-based educational resources can equalize access to correct information. Because of their level of exposure and professional standing, pharmacists have to be motivated and empowered further to carry out educational outreach.

Pharmacist-provided educational programs and materials may be a low-cost public health intervention, particularly for countries bearing the burden of the health system or care disparities regions.

From a practical perspective, the results support the role of community pharmacists as key players in women’s health education. Pharmacists are uniquely positioned to develop and disseminate evidence-based educational materials that address common patient questions and address knowledge gaps, as highlighted in previous studies on pharmacist-led patient education and pharmaceutical care [27,28]. Although this study assessed the leaflet as a standalone educational tool, incorporating such materials into routine pharmacy consultations could further enhance their impact.

## Figures and Tables

**Table 1:** Characteristics of respondents (n = 266).

Variable	Category	n	%
Age (years)	18–24	89	33.6
	25–34	109	41.1
	35–44	36	13.6
	45–54	21	7.9
	55–64	10	3.8
Education	Vocational	38	14.3
	Secondary	102	38.3
	Higher	126	47.4
Place of residence	Town ≤ 50,000 inhabitants	82	30.8
	Town 50,000–100,000 inhabitants	42	15.8
	Town 100,000–500,000 inhabitants	37	13.9
	City > 500,000 inhabitants	97	36.5
	Rural area	8	3.0

**Table 2:** Comparison of correct answers given by respondents in the pre-test and post-test survey on women’s health (n = 266).

		Knowledge Before Reading Educational Materials About Women’s Health	Knowledge After Reading Educational Materials About Women’s Health
Correct answers (%)	1. Long-acting reversible contraception	147 (55.3%)	240 (90.2%)
	2. Medication safety during pregnancy	225 (84.6%)	253 (95.1%)
	3. Pregnancy test—detection of hCG	197 (74.1%)	263 (98.9%)
	4. Interpretation of pregnancy test results	87 (32.7%)	239 (89.8%)
	5. STI self-testing and prevention	96 (36.1%)	235 (88.3%)

**Table 3:** Respondents’ self-assessment of educational materials on women’s health (n = 266).

Category	Interests	Aesthetics	Transparency	Understandability	Usability
Definitely good	48.5%	62.0%	61.7%	63.5%	61.7%
Rather good	41.7%	32.3%	29.3%	30.8%	27.8%
No opinion	4.9%	4.1%	5.6%	5.6%	9.4%
Rather poor	4.9%	1.5%	3.4%	0.0%	1.1%
Definitely poor	0.0%	0.0%	0.0%	0.0%	0.0%

**Table 4:** Respondents’ self-assessment of educational materials on women’s health by age group (n = 266).

Feature [Mean/Median]	Age [Years]	18–24	25–34	35–44	45–54	55–64	*p* Value
Interests		4.4/4.0	4.6/5.0	3.9/4.0	4.0/4.0	3.8/4.0	*p* = 0.0015
Aesthetics		4.6/5.0	4.7/5.0	4.3/4.0	4.1/4.0	4.2/4.0	*p* < 0.0001
Transparency		4.5/5.0	4.7/5.0	4.3/4.5	4.0/4.0	3.6/4.0	*p* = 0.0055
Understandability		4.6/5.0	4.7/5.0	4.4/4.0	4.2/4.0	4.1/4.0	*p* = 0.0001
Usability		4.5/5.0	4.7/5.0	4.3/4.0	4.0/4.0	4.0/4.0	*p* < 0.0001

## Data Availability

The data presented in this study are available on request from the corresponding author due to privacy and ethical reasons.

## Supplementary Materials

The following supporting information can be downloaded at: https://www.mdpi.com/article/10.3390/pharmacy14010029/s1, File S1: Educational leaflet for women’s health; File S2: Knowledge Questionnaire on Women’s Health; File S3: Questionnaire on the Evaluation of the Educational Leaflet; Figure S1: Comparison of respondents’ knowledge before and after reading educational materials on women’s health (n = 266; *p* = 0.0001); Figure S2. Dependence of the level of interest in educational materials on women’s health on the age of respondents (n = 266; *p* = 0.0015); Figure S3: The relationship between the level of interest in educational materials on women’s health and the education of respondents (n = 266; *p* < 0.0001); Figure S4: The relationship between the mean rating of understandability of educational materials on women’s health and the respondents’ education (n = 266, *p* < 0.0001); Figure S5. The relationship between the mean rating of usability of educational materials on women’s health and the respondents’ education (n = 266, *p* < 0.0001).

## References

[1] White N. (2018). Lifestyle Medicine and Health and Wellness Coaching in Pharmacy Practice. Am. J. Lifestyle Med..

[2] SW Research Ranking of Prestigious and Socially Respected Professions. https://swresearch.pl/ranking-zawodow.

[3] Supreme Pharmaceutical Chamber Report: Pharmacist in Poland. https://nia.org.pl/wp-content/uploads/2019/04/Raport_Farmaceuta_w_Polsce_2019.pdf.

[4] Tsuyuki R.T., Beahm N.P., Okada H., Al Hamarneh Y.N. (2018). Pharmacists as Accessible Primary Health Care Providers: Review of the Evidence. Can. Pharm. J..

[5] (2020). The Act on the Profession of Pharmacist. https://eur-lex.europa.eu/legal-content/PL/TXT/PDF/?uri=NIM:202106858.

[6] Polish Pharmaceutical Society Pharmaceutical Care Strategy. https://www.gov.pl/web/zdrowie/opieka-farmaceutyczna---raport.

[7] Willborn R.J. (2016). Pharmacy’s influence and opportunity in public health. Am. J. Health-Syst. Pharm..

[8] OECD, European Commission (2025). Country Cancer Profiles: Poland 2025.

[9] Polish Ministry of Health Two Modern Screening Tests for Cervical Cancer. https://www.gov.pl/web/zdrowie/dwa-nowoczesne-badania-w-profilaktyce-raka-szyjki-macicy.

[10] Statistics Poland SDG Report 2023: Women on the Path to Sustainable Development. https://raportsdg.stat.gov.pl/2023/en/Report_SDG_2023.pdf.

[11] WHC Women’s Health Barometer Report 2024: “Women in the Queue”. https://medkurier.pl/wp-content/uploads/2024/05/KOBIECY-BAROMETR-WHC-2024.pdf.

[12] Kosycarz E., Walendowicz K. (2019). Świadomość zdrowotna jako kluczowy determinant stanu zdrowia społeczeństwa. Stud. I Pr. Kol. Zarządzania I Finans..

[13] Ley P. (1988). Improving patients’ understanding, recall, satisfaction and compliance. Br. J. Clin. Psychol..

[14] Coulter A., Ellins J. (2007). Effectiveness of strategies for informing, educating, and involving patients. BMJ.

[15] Sustersic M., Gauchet A., Foote A., Bosson J.L. (2017). How best to use and evaluate Patient Information Leaflets given during a consultation: A systematic review of literature reviews. Health Expect.

[16] Giguère A., Légaré F., Grimshaw J., Turcotte S., Fiander M., Grimshaw J.M., Gagnon M.-P., Auguste D.U., Massougbodji J. (2016). Printed educational materials: Effects on professional practice and patient outcomes. Br. J. Gen. Pract..

[17] Houts P.S., Doak C.C., Doak L.G., Loscalzo M.J. (2006). The role of pictures in improving health communication: A review of research on attention, comprehension, recall, and adherence. Patient Educ. Couns..

[18] Kessels R.P.C. (2003). Patients’ memory for medical information. J. R. Soc. Med..

[19] Berkman N.D., Sheridan S.L., Donahue K.E., Halpern D.J., Crotty K. (2011). Low health literacy and health outcomes: An updated systematic review. Ann. Intern. Med..

[20] Stableford S., Mettger W. (2007). Plain language: A strategic response to the health literacy challenge. J. Public Health Policy.

[21] Dunnett J., Holkham J., Trebacz A., Baldasera C., Francis C., Dawson L., Swiers R., Christie-de-Jong F. (2025). Effectiveness and acceptability of interventions to improve readability of patient healthcare materials: A narrative systematic review. Public Health.

[22] Kelly M.P., Barker M. (2016). Why is changing health-related behaviour so difficult?. Public Health.

[23] McGuire W.J., Lindzey G., Aronson E. (1985). Attitudes and attitude change. Handbook of Social Psychology.

[24] CDC Clear Communication Index. https://www.cdc.gov/ccindex.

[25] World Health Organization (2013). Health Literacy: The Solid Facts.

[26] Redaktor Logios Configuration Documentation. https://redaktor.logios.dev/config.

[27] Harris I.M. (2024). Pharmacists’ role in combating medical misinformation. J. Am. Coll. Clin. Pharm..

[28] Slavcheva K., Staynova R., Neycheva N., Kafalova D. (2025). Community pharmacists’ knowledge, attitudes, and practices on patient education and counseling. Nutrients.

[29] Agomo C., Udoh A., Kpokiri E., Opio J.O. (2018). Community pharmacists’ contribution to public health: Assessing the global evidence base. Pharm. J..

[30] Alsheheri S.M.S., Altawyan M.S.M., Alshehri B.M. (2022). The expanding role of pharmacists in preventive health: Immunization and beyond. J. Pharma. Negat. Results.

[31] Hedima E.W., Adeyemi O., Moles R.J. (2025). Enhancing the roles of community pharmacists in primary care through continuous education and supportive policy frameworks. BMC Health Serv. Res..

[32] Aizpurua-Arruti X., Gastelurrutia M.A., Benrimoj S.I. (2024). Outcomes of community pharmacy interventions on patients with medicine use and safety issues. Integr. Pharm. Res. Pract..

[33] Afzal S., Khan F.U., Aqeel M.T., Ullah M., Bajwa M., Akhtar M., Majid M. (2023). Impact of a pharmacist-led educational intervention on knowledge, attitude, and practices regarding rational antibiotic use. Front. Pharmacol..

[34] Motlohi N.F., Makhoba X.H., Labuschagne R., Petrus R., Bangalee V. (2023). A systematic review of the role of community pharmacists in preventing and controlling cardiovascular diseases. Syst. Rev..

[35] Awadallah H., Abuiram I. (2024). The Role of Community Pharmacists in Enhancing Patient-Centered Healthcare Delivery: A Literature Review. Front. Health Inform..

