# Supplementary material for: Evaluation of Pharmacist-Developed Educational Leaflets for Women’s Health: A Pre–Post Study of Knowledge and Perceived Usefulness
Source: Pharmacy (Basel). 2026 Feb 5;14(1):29. doi: 10.3390/pharmacy14010029 (PMC12922133; doi:10.3390/pharmacy14010029)
Supplement: Supplementary file 1 [file pharmacy-14-00029-s001.zip › Figures - supplementary 1-5.pdf]

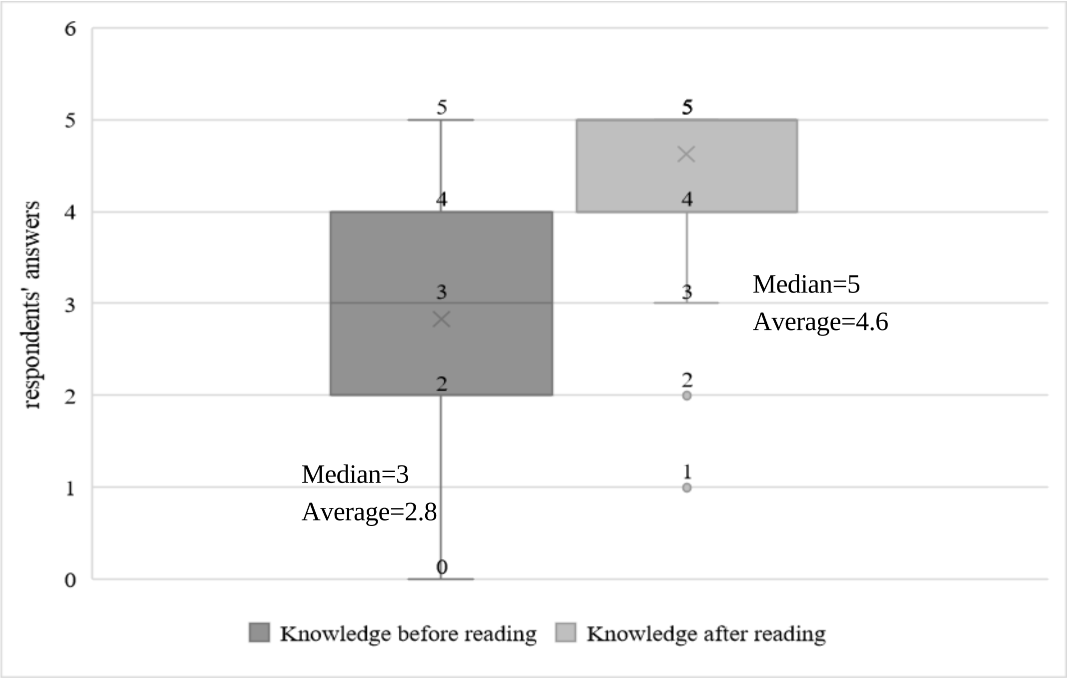

Figure S1. Comparison of respondents' knowledge before and after reading educational materials on women's health (n=266; p=0.0001)

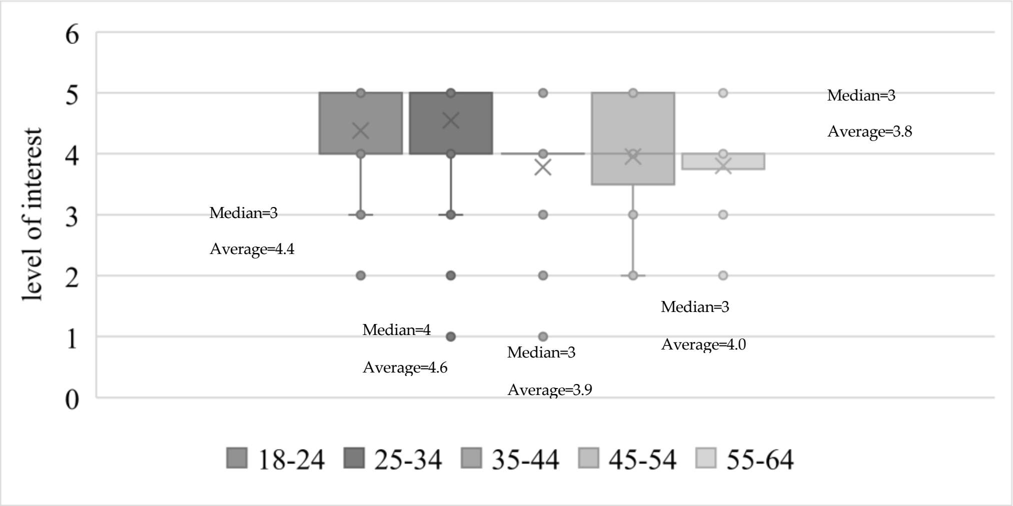

Figure S2. Dependence of the level of interest in educational materials on women's health on the age of respondents (n=266; p=0.0015)

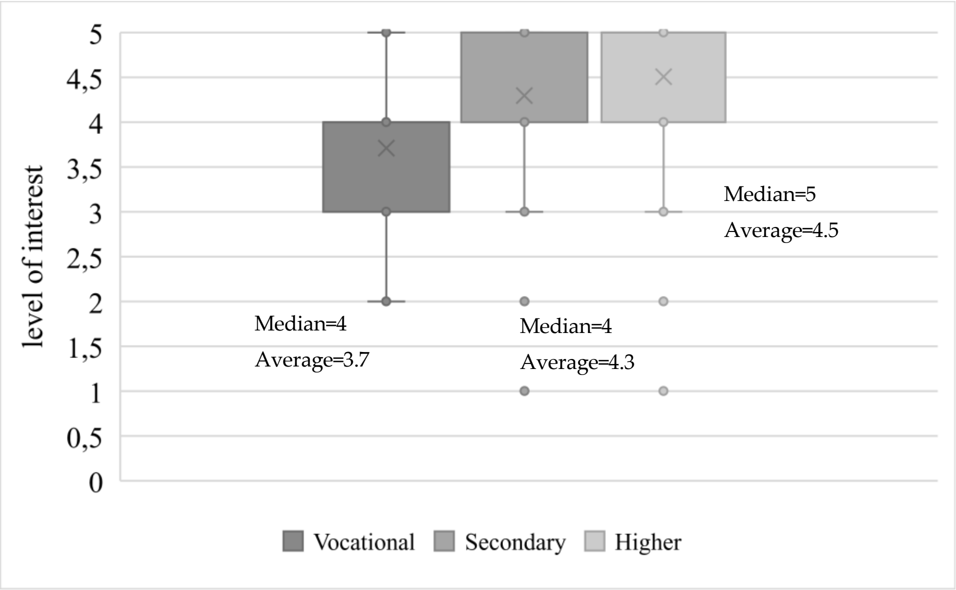

Figure S3. The relationship between the level of interest in educational materials on women's health and the education of respondents (n=266; p<0.0001)

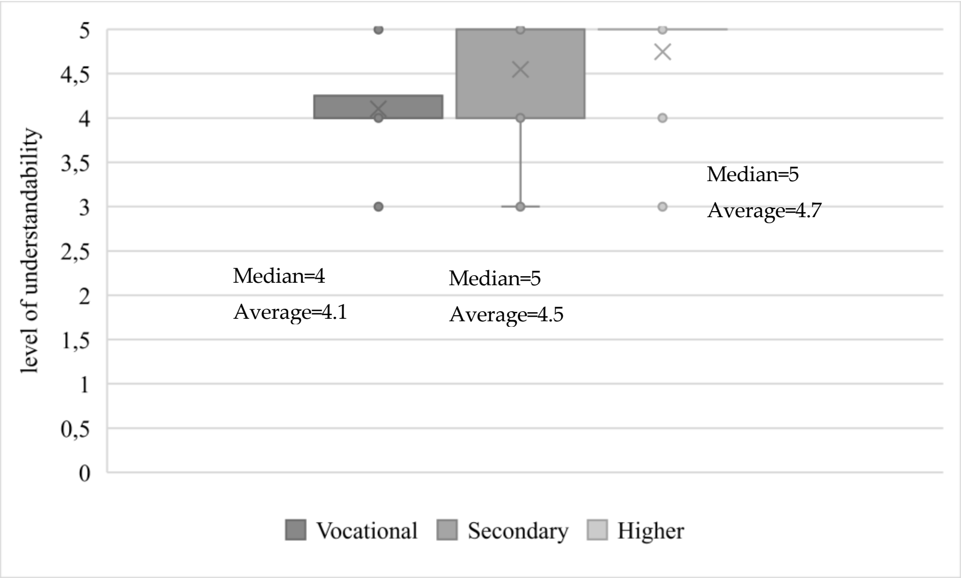

Figure S4. The relationship between the mean rating of understandability of educational materials on women's health and the respondents' education (n=266, p<0.0001)

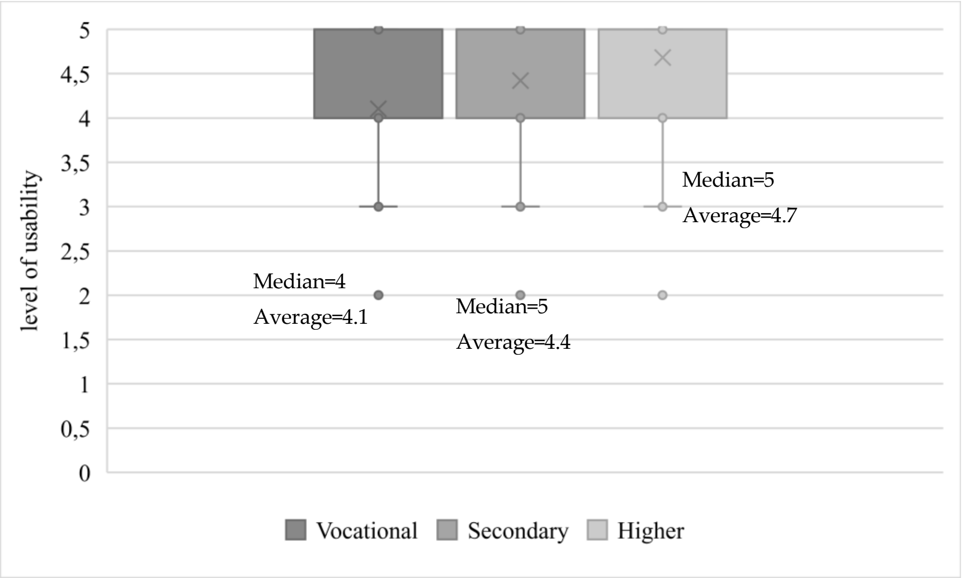

Figure S5. The relationship between the mean rating of usability of educational materials on women's health and the respondents' education (n=266, p<0.0001)
