# Supplementary material for: Evaluation of Pharmacist-Developed Educational Leaflets for Women’s Health: A Pre–Post Study of Knowledge and Perceived Usefulness
Source: Pharmacy (Basel). 2026 Feb 5;14(1):29. doi: 10.3390/pharmacy14010029 (PMC12922133; doi:10.3390/pharmacy14010029)
Supplement: Supplementary file 1 [file pharmacy-14-00029-s001.zip › File S1 pharmacy-4061556-supplementary-Educational leaflet for women’s health.pdf]

# CONVENTIONAL CONTRACEPTION

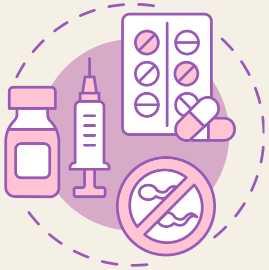

There are numerous contraceptive methods available that enable you to intentionally plan for parenthood. The selection should be customized to align with your lifestyle and health considerations. and strategies for the future.

Consult your gynecologist to select the contraceptive method that is most suitable for you.

## Hormonal contraceptives

Long-acting contraceptive methods that necessitate minimal commitment from you:
**Contraceptive implant – a slender rod inserted beneath the skin.**

- **The intrauterine device (IUD) is a compact medical apparatus placed within the uterus. A hormone-free variant is also available.**

Methods that necessitate correct and systematic usage:
**Birth control pills are hormonal oral medications taken daily, typically with a brief interval between packs.**

- **Contraceptive injection - a muscular injection that requires repetition every few weeks.**
- **The contraceptive patch is affixed to the skin for one week, after which it is substituted with a new patch. Following three weeks of use, a break is observed.**
- **The vaginal ring is a flexible disc that releases hormones and is inserted into the vagina for a duration of three weeks, followed by a one-week interval.**

# MEDICATIONS IN PREGNANCY

During pregnancy, the well-being of both the mother and the baby The health of your baby is of utmost importance. Improper use of medications can adversely affect the developing fetus and the progression of pregnancy.

## IF YOU ARE CURRENTLY ON MEDICATION...

...and if you suspect you may be pregnant, take a pregnancy test. Should the result be positive, contact your physician without delay. Your specialist will evaluate the benefits and risks associated with continuing treatment. They may suggest transitioning to a medication that is safer during pregnancy.

## IF YOU ARE ALREADY EXPECTING...

...and if you wish to use medications safely, adhere to the following guidelines:

Always seek the advice of your pharmacist or physician prior to taking any medications, including those available over-the-counter.

1. Ensure that your physician is aware of any products you are currently utilizing. The term "natural" does not always equate to "safe"—in addition to medications, dietary supplements and herbal remedies can also influence a child's development.
2. Observe your body's responses to the medications you are taking.
3. Do not independently adjust the dosages of prescribed medications.
4. Do not take medications prescribed for another individual, even if they exhibit similar symptoms and are also pregnant.
5. Read medicine labels and leaflets meticulously. Some may contain alcohol. Remember:

**There is no safe level of alcohol consumption during pregnancy.**

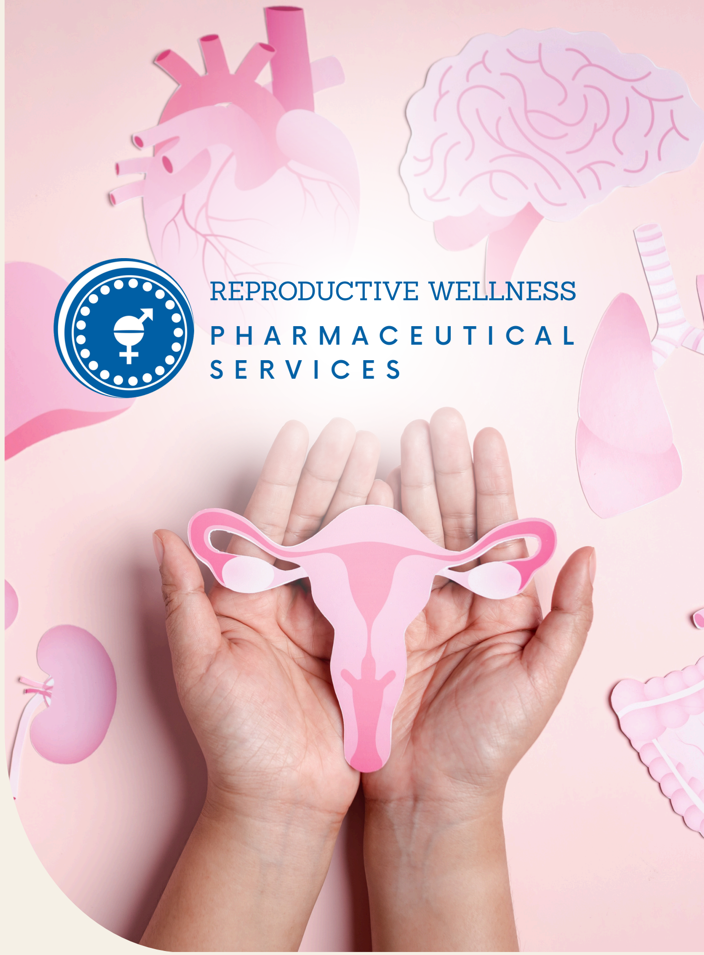

REPRODUCTIVE WELLNESS
PHARMACEUTICAL SERVICES

# HEALTH WOMEN

## TAKE CARE OF YOURSELF!

**Your health is of paramount importance to us, which is why you are receiving this leaflet. Should you have any questions or concerns regarding your intimate health or the medications you are currently taking, please do not hesitate to reach out to us.**
**- consult your pharmacist.**

# PREGNANCY ASSESSMENTS

Below, you will find information to assist you in accurately administering a pregnancy test and interpreting its results.

## HOW DOES A PREGNANCY TEST FUNCTION?

Pregnancy tests function by identifying the hormone hCG (human chorionic gonadotropin), which is generated following the implantation of a fertilized egg in the uterus. The levels of this hormone increase swiftly during the initial weeks of pregnancy.

## WHEN SHOULD ONE TAKE A PREGNANCY TEST?

The pregnancy hormone may be detected as early as 7 to 14 days following conception. However, it can occasionally take up to 21 days for a pregnancy test to confirm the presence of pregnancy.

Take a test if your period is late or at least 2-3 weeks after engaging in unprotected intercourse.

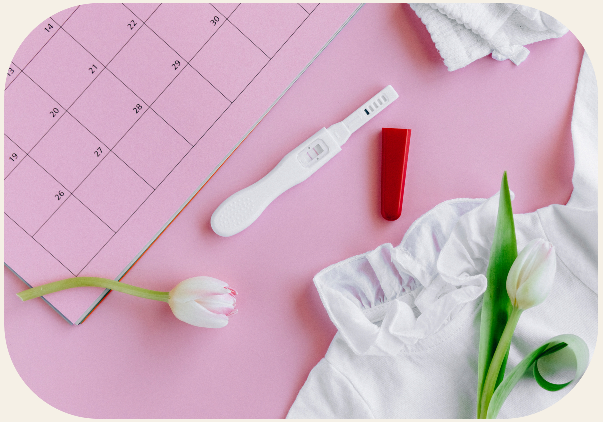

## HOW DOES ONE CONDUCT A URINE PREGNANCY TEST?

- Please read and adhere to the instructions provided with the test package.
- Typically, the optimal time to conduct a test is in the morning, as the concentration of the pregnancy hormone in your urine is at its peak immediately upon waking.
- After applying the urine sample to the test, allow the recommended duration (typically a few minutes) before interpreting the result. Never assess a pregnancy test result beyond the timeframe specified in the accompanying instructions.
- The result typically manifests as a line.

## HOW SHOULD ONE INTERPRET THE TEST RESULTS?

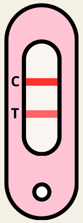


- **Positive result (two lines, test line may be faint): You are likely pregnant. Please reach out to us.**
- with a gynecologist to verify this. The physician will attend to your needs.
- and outline the steps to follow.
- False positive results are infrequent. They may arise from hormonal imbalances, specific medical conditions, and the use of certain medications.

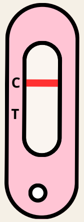


- **Negative result (line only in control zone C): This typically indicates that you are not pregnant. A false negative result may occur if the test is conducted too early.**
- **If your menstruation does not occur, repeat the test after a few days or consult your physician.**

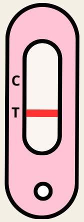


- **Invalid result (a line appears)**
- **In the test field T, but not in the control field C, the test must be repeated. Please remember the tests.**
**Pregnancy tests are highly reliable; however, If uncertain, seek guidance.**
**with a pharmacist or physician.**

# INTIMATE AILMENTS

Sexually transmitted infections (STIs) are diseases transmitted through sexual contact with an infected individual.

## INQUIRE WITH YOUR PHARMACIST REGARDING SELF-TESTING OPTIONS.

Tests are available that enable you to analyze a urine sample, perform a finger prick blood test, or assess vaginal discharge from the comfort of your home.

They can be utilized to assess vaginal pH and identify HIV (which contributes to the onset of AIDS), syphilis, chlamydia, mycosis, trichomoniasis, or Gardnerella vaginalis infection.

Verify the duration after intercourse during which a test can be conducted to detect an infection. Vaginal tests are designed for women, whereas other tests can also be administered by men.

**REMEMBER!**

- Untreated STDs can result in severe complications. If your test result is positive, consult a physician. They will verify the infection and establish the necessary treatment.
- Do not utilize a test that is expired or compromised.
- If you are apprehensive about pricking your finger or have a blood clotting disorder, it may be advisable to have the test conducted at a diagnostic laboratory.
- Home tests are beneficial; however, they do not substitute for regular evaluations by specialists.
- **Condoms significantly diminish the risk of acquiring a sexually transmitted disease.**
