# Supplementary material for: Evaluation of Pharmacist-Developed Educational Leaflets for Women’s Health: A Pre–Post Study of Knowledge and Perceived Usefulness
Source: Pharmacy (Basel). 2026 Feb 5;14(1):29. doi: 10.3390/pharmacy14010029 (PMC12922133; doi:10.3390/pharmacy14010029)
Supplement: Supplementary file 1 [file pharmacy-14-00029-s001.zip › File S2 pharmacy-4061556-supplementary-Knowledge Questionnaire on Women.pdf]

Knowledge Questionnaire on Women's Health

Dear Participant,
You are kindly invited to complete a questionnaire on women's health twice: before and after reading the educational leaflet on this topic.
The questionnaire is anonymous, and all results will be used exclusively for scientific purposes. Completing the questionnaire will take only a few minutes. Participation in this survey is voluntary. You may withdraw at any time without providing a reason. By completing the questionnaire, you confirm your informed consent to participate in the study.
Thank you for taking part in the study.

1. Choose contraceptive methods that require minimal involvement from the woman and provide long-term effectiveness (you may select more than one answer):

a) *Contraceptive implant*
b) *Intrauterine device (IUD)*
c) Vaginal ring
d) I do not know

2. Pregnant women should follow the principles below (you may select more than one answer):

a) *They should inform a doctor or pharmacist about every medication they intend to use, even if it is a herbal product*
b) They may use medications taken by other pregnant women without consulting a doctor or pharmacist
c) *They should not independently increase or decrease medication doses without consulting a doctor or pharmacist*
d) *They should not take medications that contain alcohol*
e) I do not know

3. Pregnancy test:

a) *Detects the hormone human chorionic gonadotropin (hCG)*
b) Can detect pregnancy as early as 3 days after unprotected intercourse
c) It is best performed before going to sleep
d) I do not know

4**. What does the appearance of only one line in the test (in the test area marked "T") indicate?**

a) Positive result
b) Negative result
c) *Invalid result*
d) I do not know

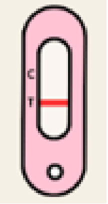

5. Please indicate whether the following statements are true or false:

a) Home tests can detect HIV infection
☐ *True* / ☐ False

b) The use of condoms reduces the risk of contracting sexually transmitted infections
☐ *True* / ☐ False

c) It is possible to perform home tests for several sexually transmitted infections
☐ *True* / ☐ False
