# Supplementary material for: Evaluation of Pharmacist-Developed Educational Leaflets for Women’s Health: A Pre–Post Study of Knowledge and Perceived Usefulness
Source: Pharmacy (Basel). 2026 Feb 5;14(1):29. doi: 10.3390/pharmacy14010029 (PMC12922133; doi:10.3390/pharmacy14010029)
Supplement: Supplementary file 1 [file pharmacy-14-00029-s001.zip › File S3 pharmacy-4061556-supplementary-Questionnaire on the Evaluation of the Educational Leaflet.pdf]

Questionnaire on the Evaluation of the Educational Leaflet

Dear Participant,
We would like to ask for your opinion on the presented educational leaflet. The survey is anonymous, and the results will be used exclusively for scientific purposes.
Please mark the appropriate answer with an **X** or write your response in the space provided. Completing the questionnaire will take a few minutes.
Thank you for taking part in the study

1. Gender:

☐ Female ☐ Male

2. Age:

…………………………………………

3. Education level:

☐ Primary
☐ Lower secondary
☐ Vocational
☐ Secondary
☐ Higher

4. Place of residence:

…………………………………………

5. Do you suffer from any chronic disease?

☐ No
☐ Yes:
– diabetes (since when?) ………………………
– asthma (since when?) ………………………
– hypertension (since when?) ………………………
– other (since when?) ………………………

6. Were you interested in the content of the leaflet?

☐ Not interested at all
☐ Rather not interested
☐ No opinion
☐ Rather interested
☐ Definitely interested

7. How do you assess the aesthetics of the leaflet (e.g., is it visually appealing)?

☐ Definitely poor
☐ Rather poor
☐ No opinion
☐ Rather good
☐ Definitely good

8. How do you assess the clarity of the leaflet (is it easy to read and understand)?

☐ Definitely poor
☐ Rather poor
☐ No opinion
☐ Rather good
☐ Definitely good

9. How do you assess the usefulness of the information provided in the leaflet?

☐ Definitely poor
☐ Rather poor
☐ No opinion
☐ Rather good
☐ Definitely good

10. How do you assess your understanding of the information provided in the leaflet?

☐ Definitely poor
☐ Rather poor
☐ No opinion
☐ Rather good
☐ Definitely good

11. How much time did you spend reading the leaflet?

………………… minutes

12. How do you assess your level of knowledge BEFORE reading the leaflet?

☐ Definitely poor
☐ Rather poor
☐ No opinion
☐ Rather good
☐ Definitely good

13. How do you assess your level of knowledge AFTER reading the leaflet?

☐ Definitely poor
☐ Rather poor
☐ No opinion
☐ Rather good
☐ Definitely good

14. Should any changes be introduced to the leaflet?

☐ No
☐ Yes, what kind?
………………………………………………………………………………

15. Any other comments or suggestions?

………………………………………………………………………………

Please return the completed questionnaire. Thank you for your time!
